# Flourishing as a Pathway to Well-Being in Obesity Care

**DOI:** 10.3390/jpm15120572

**Published:** 2025-11-28

**Authors:** Brunna Boaventura, Harold G. Koenig, Fatima Cody Stanford

**Affiliations:** 1 Department of Nutrition, Health Sciences Center, Federal University of Santa Catarina, Florianópolis 88040-370, SC, Brazil; 2 Department of Medicine, Duke University Medical Center, Durham, NC 27710, USA; harold.koenig@duke.edu; 3 Department of Psychiatry, Duke University Medical Center, Durham, NC 27710, USA; 4 Center for Spirituality, Theology, and Health, Duke University Medical Center, Durham, NC 27710, USA; 5 Department of Medicine, King Abdulaziz University, Jeddah 21589, Saudi Arabia; 6 Department of Psychiatry, Shiraz University of Medical Sciences, Shiraz 71348-45846, Iran; 7 Department of Medicine, Harvard Medical School, Boston, MA 02115, USA; fstanford@mgh.harvard.edu; 8 Department of Pediatrics, Harvard Medical School, Boston, MA 02115, USA; 9 MGH Weight Center, Massachusetts General Hospital, Boston, MA 02114, USA; 10 Department of Medicine-Neuroendocrine Unit, Massachusetts General Hospital, Boston, MA 02114, USA; 11 Department of Pediatrics-Endocrinology, Massachusetts General Hospital, Boston, MA 02114, USA; 12 Nutrition Obesity Research Center at Harvard (NORCH), Boston, MA 02114, USA

**Keywords:** obesity management, healthcare, flourish, well-being, holistic care, weight stigma

## Abstract

Obesity has increased in prevalence worldwide and is now recognized by the World Health Organization as a global epidemic. Conventional treatments remain predominantly weight-loss-oriented, and although weight is a relevant and necessary clinical indicator, relying on it alone fails to capture the full complexity of health and well-being for people living with obesity. This review proposes a comprehensive conceptual framework grounded in flourishing, advocating for a more holistic and person-centered approach to obesity care. Flourishing encompasses six key domains: life satisfaction and happiness, mental and physical health, meaning and purpose, character and virtue, close social relationships, and financial and material stability. Integrating these domains alongside traditional clinical outcomes allows obesity care to move beyond a narrow weight focus, incorporating strategies that also foster resilience, social connectedness, and purpose. This approach aligns with personalized medicine, supporting interdisciplinary and individualized care. Considering persistent weight stigma and discrimination, embracing a whole-person perspective is not optional but rather essential. By placing the individual, not only the disease or the body, at the center of care, a flourishing perspective complements biomedical indicators and offers a more compassionate and sustainable model of obesity management. However, progress will depend on the openness of healthcare, education, research, and policy stakeholders to adopt new approaches that align care with what truly constitutes well-being for people living with obesity.

## 1. Introduction

Obesity has increased in prevalence worldwide, and the World Health Organization has declared it a global epidemic [1]. Without drastic intervention, an estimated 3.8 billion adults aged 25 years and older will be living with overweight or obesity by 2050. This polycrisis will cause more adverse health outcomes in the coming decades than any other modifiable risk at an individual level [2]. The economic burden of the obesity epidemic is projected to reach USD 3 trillion annually by 2030 and exceed USD 18 trillion by 2060 [3].

Historically, healthcare providers have centered the management of obesity on weight or body mass index (BMI), which can both underestimate and overestimate adiposity. This approach provides inadequate information about individual health and undermines medically sound approaches to healthcare and policy [4]. Obesity is a condition involving excess body fat, which may or may not include abnormal distribution of fat tissue, and its causes are multifactorial and not yet fully understood [4]. While weight loss remains a relevant clinical indicator of obesity treatment, healthcare professionals must accompany it with a broader perspective that actively considers other dimensions related to the well-being of people living with obesity.

Unfortunately, weight-based bias and stigma are significant obstacles in efforts to prevent and treat obesity effectively [4]. A narrow focus on weight alone not only limits the scope of obesity care but also contributes to weight bias and stigma, reinforces negative stereotypes about individuals living with obesity, and impairs the quality of communication and discrimination in different settings, such as the workplace, education, healthcare settings, and society more generally [5,6].

Contemporary obesity care is evolving, with more effective treatment modalities and diagnostic criteria that move beyond BMI as the sole metric [4,7,8]. However, individuals with obesity continue to experience stigma in various social contexts, including in healthcare, often reporting that they are not heard by healthcare professionals who prioritize clinical outcomes over relational and emotional aspects of care [9,10].

People living with obesity frequently face discrimination and bias, are blamed for their condition, denied access to effective treatment, and subjected to repetitive, over-simplified advice such as “eat less and move more” [5,6]. This contributes to frustration, emotional exhaustion, and, in many cases, disengagement from the healthcare system. Moreover, verbal and nonverbal cues from healthcare professionals often convey moral superiority, reinforcing shame rather than empathy [9,10].

In this context, there is an urgent need to adopt personalized care strategies that genuinely place people living with obesity at the center of care. When a patient-centered perspective guides care, it becomes possible for professionals to provide compassionate care that is intentional, ethical, and respectful, understanding and assessing needs while alleviating suffering [11].

Health is arguably central to a person’s wholeness and well-being [12]. The concept of flourishing refers to a state in which multiple areas of a person’s life are good, both physically and emotionally, socially, spiritually, economically, and morally [12]. This condition reflects complete well-being, extending beyond conventional definitions of health, which are limited to the absence of disease [12]. In fact, for most patients, health is not experienced solely through normal laboratory values or symptom control. They are also concerned with leading meaningful lives, experiencing positive emotions, maintaining close and supportive relationships, and acting in ways that align with their values [13].

Flourishing offers a more comprehensive approach to evaluating health, considering the person across multiple domains that impact well-being [12]. Unlike traditional healthcare measures, it captures neglected aspects of human experience relevant to clinical care. By incorporating elements of flourishing into healthcare, including obesity care, professionals may better equip themselves to support individuals as they navigate complex treatment decisions, consider different goals, and reflect on what truly matters to the patients. This review can also serve as a guide for professionals seeking to align their practice with more personalized and meaningful models of care, focusing on the person’s health rather than the body’s [13].

Although evidence on the relationship between obesity and flourishing remains limited, individuals with obesity seem to exhibit lower flourishing than those with normal weight or overweight, suggesting that strengthening flourishing related psychosocial resources may be a therapeutic target [14]. Childhood obesity is also associated with lower levels of flourishing markers, including those related to school performance and coping skills, such as completing homework, showing interest in learning, finishing tasks, staying calm when challenged, and caring about academics [15]. The COMPASS study reported that students who perceived themselves as overweight exhibited lower flourishing [16]. Coordinated efforts from schools and healthcare providers may help address these gaps and enhance overall well-being. Furthermore, understanding flourishing and the role of family, school, and community in influencing factors that contribute to childhood obesity is vital to preventing and mitigating this growing pandemic [17].

The Global Flourishing Study, a large-scale longitudinal project involving over 200,000 participants from 22 culturally and geographically diverse countries, expands our understanding of the distribution and determinants of well-being, providing foundational knowledge for promoting flourishing [18]. Despite the first wave of data revealing both commonalities and divergences in patterns of flourishing among nations, findings indicate that younger adults report lower levels of flourishing compared with older adults, suggesting age-related differences in well-being, suggesting age-related differences in well-being [18]. Economic development may also compromise meaning, relationships, and spirituality, thereby raising the need to rethink how societies promote well-being [18].

When applied to medicine, including obesity care, assessing and considering flourishing domains may provide a broader evaluation of outcomes related to the health of the person, not only in terms of physical and mental health but also the person’s overall dimensions of life, which directly and indirectly impact well-being [19]. Although this approach may be challenging in clinical decision-making, it aligns closely with the core principles of patient-centered care, being a foundation for more responsive and respectful care [13]. Many of the most critical decisions in healthcare intersect with more profound questions about identity, connection, and purpose. When care is limited to the body, it may inadvertently overlook or even conflict with other priorities central to the patient’s sense of self [13].

This review proposes a comprehensive conceptual framework that advocates for a paradigm shift in obesity care, embracing a human flourishing perspective to promote sustainable well-being and improve the overall quality of life for people living with obesity.

## 2. Methods

This narrative (non-systematic) review, guided by a comprehensive conceptual framework, synthesized evidence on flourishing in the context of obesity care by integrating theoretical, measurement, and empirical domains across disciplines and age groups. Given the heterogeneity of concepts, measures, and study designs, a narrative approach was judged more appropriate than a systematic review or quantitative meta-analysis. Literature was identified in MEDLINE/PubMed and Scopus, limited to peer-reviewed English-language publications from 2000 to 2025, with searches conducted from April to October 2025. Searches used the core keywords “flourishing AND obesity” and “flourishing AND health” applied to titles, abstracts, and keywords. In addition, the search was extended with domain-specific strategies aligned to flourishing dimensions, using obesity-linked terms tailored to each database, including life satisfaction and meaning/purpose (“obesity” AND “life satisfaction” OR “meaning in life” OR “purpose in life”), mental and physical health (“obesity” AND “mental health” OR “depression” OR “anxiety” OR “health”), character and virtue (“obesity” AND “character strengths” OR “virtue”), social relationships (“obesity” AND “social relationships” OR “social support” OR “loneliness”), and financial/material stability (“obesity” AND “financial stability” OR “income” OR “employment”). Eligible records included theoretical papers, reviews, and empirical studies with cross-sectional, longitudinal, or interventional designs in pediatric and adult populations within clinical or community settings. Non-peer-reviewed materials, non-English records, and studies without a human focus were excluded. Reference lists and forward citations were screened to identify additional sources. Evidence was organized thematically and summarized narratively.

## 3. Multidimensional Perspective of Flourishing

Flourishing, as a construct, is rooted in positive psychology, notably through Seligman’s PERMA model, which articulated well-being as comprising positive emotion, engagement, relationships, meaning, and accomplishment [20,21]. Over the past two decades, this conceptualization has been further developed and refined, with VanderWeele advancing a multidimensional framework encompassing six domains of well-being—life satisfaction and happiness, mental and physical health, meaning and purpose, character and virtue, close social relationships, and financial and material stability—and introducing validated tools for its measurement in diverse populations (Table 1) [12]. In clinical practice, the score for each domain could provide a useful indicator for identifying areas that may require further exploration. This information can guide the healthcare professional in conducting more in-depth and individualized discussions with the patient, ensuring that the care plan addresses the patient’s unique needs and values.

Altogether, this multidimensional perspective of flourishing contributes to a more nuanced understanding of health by broadening its scope beyond strictly biomedical outcomes [19]. Traditional healthcare approaches have often prioritized the health of the body, focusing on physical metrics and disease control. On the other hand, the flourishing model shifts attention toward the person’s health, incorporating the full range of human experience into the concept of well-being [13]. This is particularly relevant for the care of individuals living with chronic and stigmatized conditions such as obesity, where clinical indicators alone often fail to reflect patients’ priorities or address the cumulative impact of social, emotional, economic, and psychological stressors. Applying the human flourishing approach in clinical settings, which presents a holistic perspective regarding care, opens pathways to more personalized and compassionate care in obesity treatment, potentially revealing overlooked mechanisms in obesity and supporting more effective, comprehensive strategies for prevention and treatment [22].

## 4. Relating Flourishing to the Context of Obesity Care

**Figure 1:**
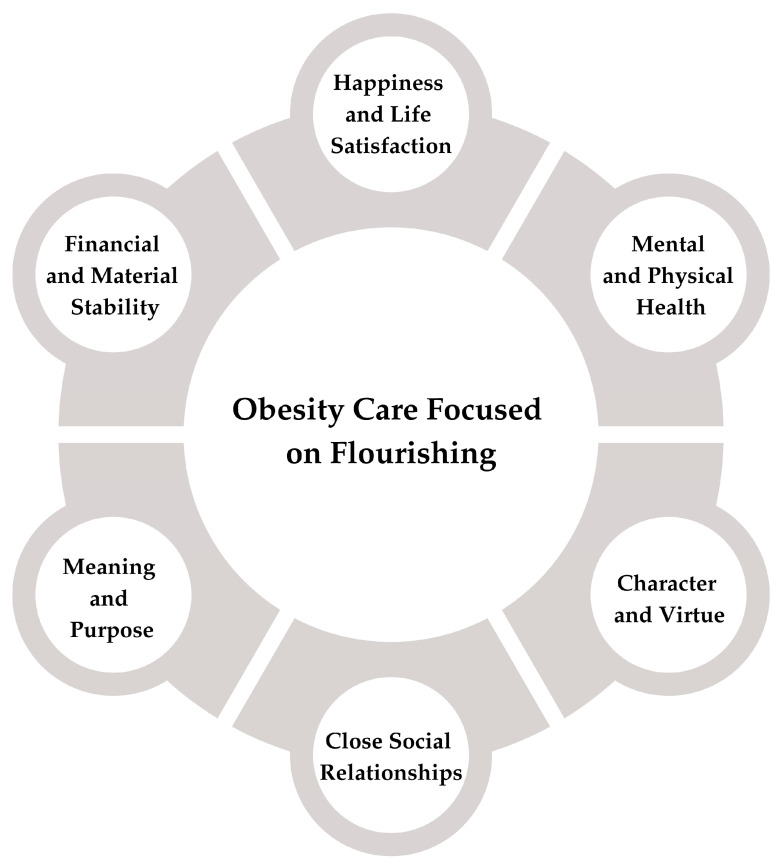
Domains of flourishing in the care of patients with obesity.

Considering the proposed flourishing domains [12], each can be related to the experiences of individuals living with obesity, highlighting the need to move beyond a narrow focus on clinical outcomes, such as weight loss, and toward a multidimensional approach to their care and well-being. Figure 1 illustrates the domains of flourishing to be considered when providing care for patients with obesity.

### 4.1. Life Satisfaction and Happiness

People living with obesity, particularly due to weight-related stigma, discrimination, internalized weight bias, and the psychological burden of the disease, experience feelings of low self-esteem, guilt, and shame, often leading to diminished feelings of life satisfaction and happiness [23,24].

Life satisfaction, happiness, positive affect, vitality, self-esteem, self-acceptance, and optimism are aspects of psychological well-being that tend to be lower in women with obesity [25]. A recent study examined the determinants of weight status, body image, health, and life satisfaction in young adults [26]. Among nearly 20% of participants classified as having overweight or obesity, excess weight significantly reduced body and health satisfaction [26]. Additionally, a study investigating the influence of BMI on health complaints and life satisfaction, provided compelling evidence that BMI plays a critical role in adolescents’ well-being and their interactions with peers at school [27].

Therefore, it is essential to investigate aspects of people living with obesity that interfere with life satisfaction and happiness, seeking to identify the impact of the disease in this domain.

### 4.2. Mental and Physical Health

Obesity is a chronic, multifactorial disease that impacts both physical and mental health. It significantly increases the risk of cardiometabolic conditions, musculoskeletal disorders, and certain cancers, primarily through mechanisms involving excess adiposity, systemic inflammation, and hormonal dysregulation [4,8]. It is also associated with impaired cognition and reduced psychological well-being, as individuals living with obesity frequently experience depression, anxiety, emotional dysregulation, impaired quality of life, body dissatisfaction, and low self-esteem [4,8]. Weight stigma and repeated treatment failures often amplify these mental health effects. Internalized stigma, stress, social isolation, and substance abuse allow weight stigma to adversely affect mental and physical health beyond the impact of obesity itself [4]. In clinical settings, weight stigma can also compromise the quality of care, leading to avoidance of medical services, delays in treatment, and reduced adherence to recommendations [5].

Notably, the COMPASS study showed that students who perceived themselves as “overweight” and had experienced bullying and victimization reported higher anxiety and depressive symptomatology and lower flourishing, compared with students who perceived their weight as “about right” and had not experienced bullying victimization [16].

In this context, the clinical understanding of obesity must extend beyond its physiological effects to encompass its substantial impact on psychological well-being and quality of life, highlighting the need for integrated, patient-centered approaches to care to enable the person to flourish.

### 4.3. Meaning and Purpose

Questions such as “Overall, to what extent do you feel the things you do in your life are worthwhile?” or “Do you understand your purpose in life?” are rarely addressed in clinical settings but are related to health and well-being. Research has identified that possessing a high sense of purpose in life reduces the risk of all-cause mortality and cardiovascular events [28]. In this context, life meaning and purpose are potentially modifiable factors that may lead to downstream health benefits. A study also demonstrated that in adults older than 50 years, a stronger life purpose is associated with lower all-cause mortality [29].

On the other hand, data on adolescents with obesity reveal a significant association between obesity and increased rates of suicidal ideation, planning, and attempts among US adolescents, even after adjusting for confounding psychosocial factors such as weight stigma and discrimination [30]. These findings show the relevance of addressing the domain of meaning and purpose in life within this population, particularly considering the mental health challenges and psychosocial distress frequently experienced by individuals living with obesity.

Therefore, it is essential to emphasize the domain of meaning and purpose in life in obesity care, as numerous factors, such as mental health, social dynamics, discrimination, and internalized stigma, may influence it and require individualized attention and appropriate intervention.

### 4.4. Character and Virtue

Character strengths such as kindness, generosity, and altruism may promote health by enhancing positive emotions and improving social cooperation, which supports adaptation to environmental changes [31].

The internalization of weight bias has been linked to poorer psychological outcomes among individuals living with obesity, often reinforcing patterns of self-criticism and undermining a stable sense of self-worth [23,24,32]. These dynamics can interfere with the cultivation of character strengths, such as self-compassion, perseverance, and integrity, which are central to the domain of character and virtue within the flourishing framework. Promoting these qualities through interventions that encourage acceptance and values-based action may play a key role in supporting psychological well-being and restoring a sense of moral agency in the face of stigma and chronic adversity [32].

### 4.5. Close Social Relationships

Social relationships represent a critical dimension of the lived experience of individuals with obesity [5]. Stigma and discrimination are consistent and, in some instances, occur daily in many social settings, leading to mental and physical health concerns, social disengagement, and dysfunctional relationships with significant others, as well as avoidance of health-promoting activities [33].

Weight stigma can undermine a person’s sense of connection and social belonging, often leading to isolation and strained relationships [34]. Evidence suggests that many people living with obesity encounter relational strain, characterized by judgment, exclusion, or a lack of understanding, across various social contexts, including healthcare settings, workplaces, and family environments [33]. These experiences often contribute to internalized stigma, reduced trust, and social withdrawal, ultimately undermining emotional well-being [24]. Anticipation of negative social evaluation may lead to avoidance of interpersonal engagement, restricting access to supportive networks [5,33].

As social relationships are central to psychosocial well-being, addressing weight-related self-stigma and fostering supportive social environments may be key to mitigating the relational harms caused by weight stigma [24,35]. Given the established link between relational connectedness and psychological health, addressing the social challenges faced by individuals with obesity is essential for promoting more comprehensive and person-centered care.

### 4.6. Financial and Material Stability

Financial and material stability is a foundational domain of flourishing, enabling individuals to meet basic needs and engage with other well-being aspects [12,18]. Unfortunately, weight stigma contributes directly to this burden by limiting access to education, employment, and career advancement. People with obesity are systematically disadvantaged in hiring practices, often receive lower wages, and face greater obstacles to professional progression [5]. For people living with obesity, concerns related to financial insecurity, such as difficulties affording food, housing, or healthcare, can intensify health vulnerabilities and limit access to effective treatment. Overweight and obesity are associated with poorer labor market outcomes for young adults, regardless of qualifications, indicating long-term economic disadvantages and the need for broader public health and policy responses [36].

People living with both food insecurity and obesity face overlapping challenges, including limited access to nutritious and healthy food, emotional distress, and harmful home dynamics [37]. Evidence from a recent systematic review shows that this paradox, where excess body weight coexists with micronutrient deficiencies, reflects structural and socioeconomic barriers that shape dietary patterns and health behaviors, often leading to higher consumption of energy-dense, nutrient-poor foods [37]. Addressing this dual burden requires integrating obesity management with strategies that improve food security and prioritize nutritional quality alongside energy balance. Additionally, these challenges can often be associated with socioeconomic disadvantages and the high cost of long-term obesity management, including medications, consultations, and lifestyle interventions [38].

These intersecting issues underscore the importance of addressing food security as a fundamental social determinant of health through comprehensive, system-level policy interventions in obesity management. Addressing material constraints is essential for delivering equitable and sustainable obesity care, particularly in populations disproportionately affected by both obesity and poverty.

## 5. Integrating Flourishing into Personalized Obesity Care

Bringing the concept of flourishing into the field of personalized medicine, including obesity care, underscores the importance of caring for the whole person, not just the disease [13,19]. While conventional healthcare already seeks to consider individual differences in genetics, behavior, and environment, flourishing broadens this scope by including elements such as meaning, virtue, relationships, social, and material conditions, dimensions often neglected in routine clinical encounters but essential to long-term well-being [13,19]. By systematically exploring each domain of flourishing, healthcare professionals are better equipped to build rapport and trust, elicit a comprehensive understanding of the patient’s life context, and appreciate their motivations and challenges, even when these stem from complex or morally difficult circumstances. This model also considers the principles of compassionate care, emphasizing the importance of recognizing the patient beyond their disease, validating their dignity, and fostering a therapeutic alliance that supports both health outcomes and overall well-being [11].

Additionally, flourishing domains vary meaningfully across demographic and contextual factors [18]. For example, financial and material stability emerged as particularly salient in lower-income populations, while domains such as meaning and purpose varied across life stages, with distinct patterns among younger and older adults [18]. Cultural differences were also observed, influencing the relative importance and expression of specific domains, such as relationships, pro-social behaviors, and community belonging [18]. These findings reinforce the need for culturally and contextually sensitive applications of flourishing-oriented frameworks in obesity care, ensuring that interventions address the most relevant domains for each population group.

To translate this into action, health professionals must develop a more holistic lens in their clinical practice [22]. That does not mean overloading already busy health consultations, but rather being intentional in asking the right questions, which can help identify what matters most to the patient. Even brief screening questions, such as the ones proposed and described in Table 1, can offer meaningful insights when integrated early in the care process [12].

When more complex and profound needs arise, referral to professionals from other areas becomes crucial. In this way, interdisciplinary approaches may shed light on the missing pieces of the obesity puzzle. Depending on individual needs, they can open new avenues for interventions at the individual or population level, including professionals such as physicians, nurses, dietitians, psychologists, physical educators, social workers, and even spiritual or financial counselors [22]. Supporting flourishing means recognizing that well-being in obesity care is built not only through the amount of weight lost or other physiological outcomes but also by considering dignity, purpose, and sustainable life conditions.

Certain activities have been linked to improvements in domains related to flourishing, encompassing cognitive, behavioral, and relational practices [39]. Cognitive activities, such as practicing gratitude, savoring positive experiences, and imagining the best possible self, have been shown to have benefits in happiness, life satisfaction, improved sleep, and reduced depressive symptoms. Behavioral activities, including using character strengths, performing acts of kindness, and volunteering, contribute not only to happiness, life satisfaction, and reduced depressive symptoms but also to enhanced social connection, increased engagement, and improved perceived health. Relationship-based and institutional activities, such as marital therapy, work and job crafting, and participation in religious communities, have demonstrated effects on meaning, life satisfaction, engagement, and longevity, with decreased depressive symptoms and suicide. Additionally, structured resources like recovery workbooks targeting depression, anxiety, and forgiveness offer support for psychological distress and emotional regulation. Together, these approaches demonstrate the range of strategies available to support flourishing in clinical and community settings. Specifically in obesity, even modest physical activity or sports participation among adolescents with obesity is associated with greater flourishing and academic engagement, extending evidence that these outcomes are typically lower than in healthy-weight peers [40].

It is noteworthy that obesity treatment encompasses behavioral modifications related to lifestyle, pharmacological treatment, and/or metabolic or bariatric surgery [8]. Considering the flourishing perspective in obesity care is not about denying proper treatment of the disease or the associated conditions, but rather about considering broader characteristics and outcomes that people living with obesity need beyond weight loss and encouraging more profound and complex aspects that interfere with their well-being. Holistic care, as proposed by the flourishing approach, does not neglect physical and physiological determinants of health but rather considers the impact of non-traditional variables, such as life purpose, social connections, and happiness, in healthcare, which in turn affects the well-being of people living with obesity.

Incorporating a flourishing perspective and encouraging activities that promote flourishing may also positively impact patient motivation and engagement. Adherence tends to improve when individuals feel that their values and goals are part of the care plan. Instead of reinforcing a cycle of prescriptions and frustrations, the conversation shifts toward building coping strategies, resilience, and more realistic health trajectories. Flourishing, in this sense, strengthens the foundations of truly compassionate and person-centered obesity care.

## 6. Barriers, Gaps, and Future Directions

Implementing a flourishing-based approach in obesity care remains limited by structural, educational, and epistemological barriers. Health systems continue to prioritize biomedical endpoints, particularly weight-related outcomes, often neglecting the multidimensional nature of well-being. Professional healthcare training frequently fails to incorporate frameworks that account for care’s psychological, social, and existential dimensions, resulting in a limited clinical focus. A key limitation could be the underrecognition of flourishing as a legitimate construct in obesity care, with challenges in integrating broader concepts, such as meaning, virtue, and relational health, into clinical practice.

Although this framework was developed in the context of obesity, its principles are potentially applicable to other chronic conditions, such as hypertension, cancer, and diabetes, where long-term management and quality of life are central goals. In such contexts, the degree of coordination among healthcare professionals may vary, but an interdisciplinary approach can enhance the integration of flourishing into patient care.

Additionally, research gaps exist regarding primary or secondary flourishing outcomes in clinical trials of obesity management, which limits the evidence base needed to guide practice and policy.

Engaging policymakers in adopting flourishing-oriented strategies is also crucial, particularly to address systemic constraints such as brief clinical encounters that may limit the depth of patient engagement.

Future directions should include incorporating flourishing-informed metrics into clinical trials, the development of interdisciplinary educational curricula, and promoting policies that support holistic, science-driven approaches to obesity management.

## 7. Conclusions

Considering obesity care from the perspective of flourishing offers a meaningful shift from a conventional, weight-focused approach to more comprehensive care, which considers health within the broader context of human well-being, encompassing domains such as life purpose, social relationships, character, and material stability, elements often overlooked in conventional clinical frameworks. Rather than reducing progress in obesity treatment to numerical outcomes, flourishing focuses on what individuals value most.

This reconceptualization of obesity care is not merely theoretical—it has practical implications for reshaping obesity treatment goals, utilizing non-stigmatizing and non-judgmental communication, and informing person-centered metrics that reflect the complexity of living with obesity. It also underscores the need to restructure healthcare professional education and healthcare systems to adopt a more holistic approach.

Considering persistent weight stigma and discrimination, embracing a whole-person framework in obesity care is not optional; it is essential. By placing the human being, rather than the body or the disease, at the center of care, a flourishing perspective offers a compassionate way of obesity management. However, the path forward will require openness from healthcare, education, research, and policy stakeholders to new modes of obesity treatment and a sustained effort to align care with what well-being truly means for people living with obesity.

## Figures and Tables

**Table 1:** Flourishing domains and measurement aspects *.

Domains	Measurement Questions **
Happiness and Life Satisfaction	Overall, how satisfied are you with life as a whole these days? In general, how happy or unhappy do you usually feel?
Mental and Physical Health	In general, how would you rate your physical health? How would you rate your overall mental health?
Meaning and Purpose	Overall, to what extent do you feel the things you do in your life are worthwhile? I understand my purpose in life.
Character and Virtue	I always act to promote good in all circumstances, even in difficult and challenging situations. I am always able to give up some happiness now for greater happiness later.
Close Social Relationships	I am content with my friendships and relationships. My relationships are as satisfying as I would want them to be.
Financial and Material Stability	How often do you worry about being able to meet regular monthly living expenses? How often do you worry about safety, food, or housing?

* Adapted with permission from: VanderWeele TJ, McNeely E. Reimagining Health—Flourishing. JAMA. 2019;321(17):1667–1668. Copyright © 2019 American Medical Association. All rights reserved. Permission License Number: 6142591060575. ** Responses to each question or statement are scored from 0 (lowest) to 10 (highest) [12].

## Data Availability

No new data were created or analyzed in this study. Data sharing is not applicable to this article.
